# Molecular diagnostics in pancreas transplantation: past, present, and future

**DOI:** 10.3389/fmolb.2026.1825364

**Published:** 2026-06-22

**Authors:** Tim D. A. Swaab, Charlotte C. A. Franken, Robert A. Pol, Maria José Ramirez-Bajo, Pedro Ventura-Aguiar

**Affiliations:** 1 Laboratori Experimental de Nefrologia i Trasplantament (LENIT), Fundació de Recerca Clinic Barcelona-Institut d’Investigacions Biomèdiques August Pi I Sunyer (FRCB-IDIBAPS), Barcelona, Spain; 2 Department of Surgery, Division of Organ Donation and Transplantation Surgery, University Medical Center Groningen, Groningen, Netherlands; 3 Redes de Investigación Cooperativa Orientadas a Resultados en Salud (RICORS 2040), Madrid, Spain; 4 Nephrology and Kidney Transplant Department, Hospital Clínic Barcelona, Barcelona, Spain; 5 Department of Medicine, Faculty of Medicine and Health Sciences, University of Barcelona, Barcelona, Spain

**Keywords:** diabetes, immunology, NanoString, pancreas transplantation, RNAseq, transcriptomics

## Abstract

Pancreas transplantation is the gold standard endocrine replacement therapy for patients with type 1 diabetes (T1D) and chronic kidney disease. Histological assessment remains the cornerstone for diagnosing pancreas graft rejection, relying on the Banff classification to standardize interpretation. Transcriptomic analyses have transformed transplantation research by enabling gene expression profiling within allografts. Studies utilizing bulk RNA (RNA-seq) and single-cell RNA sequencing (scRNA-seq) have advanced understanding of immune-mediated graft injury. However, pancreas transplantation research remains limited by small single-center cohorts, while extrapolation from other solid organ transplants is constrained by the unique structural and functional characteristics of the pancreas graft. These challenges partially arise from the distinct compartments involved in major pancreas allograft pathologies, including exocrine injury in alloimmune-rejection response and endocrine islet injury in a myriad of post-transplant beta cell stressors (i.e., T1D recurrence, CNI-toxicity, or metabolic syndrome). The application of molecular diagnostics in pancreas transplant biopsies has been demonstrated, including clinical-grade microarray technology. Nevertheless, pancreas-specific gene expression markers remain underexplored, and robust evidence regarding their clinical utility is lacking. This review conceptualizes pancreas allograft injury as a dual-compartment process involving both exocrine rejection and endocrine islet injury, thereby providing an interpretive framework for evaluating emerging molecular diagnostics in pancreas transplantation.

## Introduction

Type 1 diabetes (T1D) is characterized by immune-mediated destruction of pancreatic beta cells, leading to insulin deficiency and the need for exogenous insulin therapy (Atkinson et al., 2013). In patients with concomitant T1D and advanced chronic kidney disease (ACKD), kidney-pancreas transplantation is regarded as the “gold standard” for endocrine replacement therapy, restoring endogenous insulin secretion and physiological glucose homeostasis (White et al., 2009). Since the first successful pancreas transplant in December 1966, outcomes have significantly improved, driven by advances in surgical techniques, preservation methods, and immunosuppression (Boggi et al., 2021). However, pancreas transplant rejection (both acute and chronic) remains the leading cause of pancreatic graft loss beyond the first 3 months post-transplant (Gruessner and Gruessner, 2022). While pancreas biopsy is currently the only definitive method to confirm the diagnosis of rejection, conventional histology is limited by its descriptive nature and offers limited insight into the underlying pathogenic mechanisms (Luan et al., 2009a).

Molecular diagnostic techniques have rapidly evolved over the past 3 decades and can elucidate the mechanisms and pathways underlying specific disease processes, identify molecules suitable for non-invasive diagnostics, and reveal novel therapeutic targets. The invention of polymerase chain reaction (PCR) in 1983 enabled next-generation sequencing (NGS) and made high-throughput RNA sequencing increasingly accessible and cost-effective (Satam et al., 2023). This transformation has revolutionized transplantation research, providing detailed insights into immune cell functions and their interactions with transplanted organs.

**FIGURE 1:**
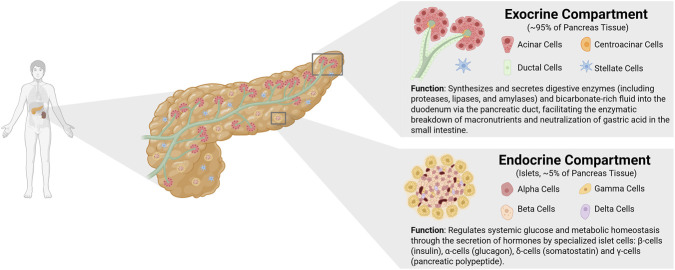
Graphic representation of major tissue compartments of the pancreas graft.

**FIGURE 2:**
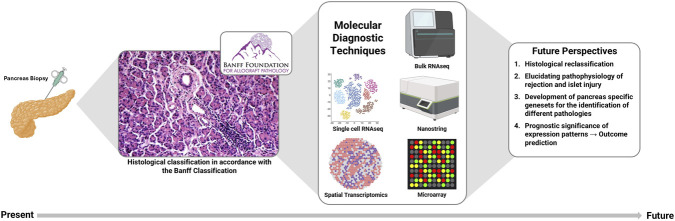
Potential molecular diagnostic techniques and future perspectives in pancreas transplantation.

Studies utilizing bulk RNA sequencing, either in tissue or plasma, have successfully identified molecular biomarkers that may refine the diagnosis of graft rejection (Mueller et al., 2024; Shah et al., 2024) and predict disease onset (Archer et al., 2022; Zhang et al., 2019), reducing the need for invasive procedures (Jeon et al., 2020) and supporting treatment decisions (Hruba et al., 2023). While there is a large degree of overlap in the mechanisms involved in allograft rejection across organs, unique organ features associated with cell type, function, and immunogenicity hinder generalization across transplanted organs (Vaulet and Naesens, 2024). The pancreas consists of exocrine and endocrine compartments, both of which may be vulnerable to injury following transplantation (Figure 1) (Drachenberg et al., 2024)^.^ These unique features of pancreas tissue highlight the necessity of molecular techniques to further elucidate the pathogenic mechanisms of rejection, including islet cell autoimmunity and rejection-induced islet injury. Translation of these molecular diagnostic techniques from ‘bench to bedside’ may ultimately enable non-invasive graft monitoring and improve pancreas transplant outcomes (Figure 2).

This mini-review explores the current state of molecular diagnostics research in pancreas transplantation. Although the existing evidence remains limited and largely exploratory, emerging molecular diagnostic approaches show promise for refining the diagnosis and classification of pancreas graft rejection and for improving understanding of islet injury in the context of rejection.

## Transcriptomics in organ transplantation

Graft rejection is a major challenge in organ transplantation, potentially leading to graft dysfunction or failure. The introduction of the Banff classification was instrumental in standardizing criteria for assessing transplant pathology (Loupy et al., 2022). In addition to being the diagnostic gold standard, the classification has been pivotal in driving the transition from histology-based diagnosis to molecular diagnostic approaches (Mengel et al., 2020). Identifying specific molecular expression patterns in allograft biopsies associated with various types of allograft injury may elucidate mechanisms underlying transplant rejection or dysfunction and facilitate the development of molecular classification scores (Loupy et al., 2022; Mengel et al., 2020; Hruba et al., 2025)^.^

Early molecular diagnostic studies in renal allograft rejection primarily focused on acute T-cell–mediated rejection. Using reverse transcription polymerase chain reaction (RT-PCR) to quantify intra-graft immune activation gene expression, these studies demonstrated a strong correlation between elevated cytokines, Fas ligand, perforin, granzyme B, and acute renal allograft rejection (Strehlau et al., 1997; Desvaux et al., 2004). Over time, the use of DNA microarrays to analyze gene expression patterns gained prominence, enabling the subtyping of acute rejection based on distinct immunologic and cellular characteristics and offering new insights into the molecular mechanisms driving graft rejection. Notably, the presence of B-cell markers in acute rejection has received increasing attention due to their strong association with severe graft dysfunction (Sarwal et al., 2003).

Pivotal studies from the University of Alberta used gene expression data to detect antibody-mediated rejection (ABMR) and T-cell–mediated rejection (TCMR) in 403 renal transplant biopsies (Sellarés et al., 2013; Reeve et al., 2013). Acknowledging the limitations of traditional diagnostic features for ABMR, they developed a microarray-based molecular classifier using the 30 most differentially expressed genes, which could independently assess the probability of ABMR in transplant biopsies regardless of histology, donor-specific antibodies (DSAs), or C4d staining (Sellarés et al., 2013), and these findings contributed to the introduction of diagnostic criteria for C4d-negative ABMR at the 2013 Banff meeting (Haas et al., 2014). Simultaneously, they created a molecular classifier for TCMR utilizing the same method enabling differentiation between TCMR and other biopsy types (Reeve et al., 2013).

An additional important advancement was the introduction of the Molecular Microscope Diagnostic System (MMDx), the first integrated diagnostic system of its kind (Reeve et al., 2019). This system combines genome-wide microarrays to measure the expression of 49,495 probe sets corresponding to 19,462 genes in transplant biopsies and uses machine learning algorithms to generate comprehensive diagnostic reports. Since its initial development, MMDx has undergone significant refinement and has emerged as a valuable tool in clinical practice, improving diagnostic precision and being often used in clinical trials as an add-on marker of treatment response (Mayer et al., 2024).

As gene expression analysis became increasingly recognized as a diagnostic tool for allograft rejection, the 2017 Banff meeting report identified key genes for diagnosing TCMR and ABMR, further supporting its clinical adoption (Haas et al., 2018). Two years later, at the 2019 Banff meeting, the Banff Molecular Diagnostics Working Group (MDWG) introduced a novel multi-organ transplant gene panel (Mengel et al., 2020). The Banff Human Organ Transplant (B-HOT) gene set, comprising 770 genes linked to injury, innate and adaptive immunity, and rejection. The genes were selected based on available literature describing gene expression across various injuries and insults in transplanted organs. The B-HOT panel offers a significant advantage by enabling cross-study comparisons and facilitating the identification of shared rejection pathways across different organs. Studies applying the B-HOT panel have demonstrated its utility and feasibility in exploring gene set scores correlating with disease (Beadle et al., 2023; Giarraputo et al., 2023).

Single-cell molecular studies represent a step forward in the characterization of alloimmune rejection. Recent reviews highlight how single-cell sequencing technologies allow high-resolution characterization of immune cell states and intercellular communication networks driving graft rejection and tolerance (Mou and Pu, 2025). Previously, in a landmark study using droplet-based single-cell RNA sequencing (scRNA-seq), the Leuven group developed an immune cell atlas of kidney graft rejection. A strong correlation between FCGR3A + monocytes, natural killer (NK) cells, and intra-graft inflammation was identified (Lamarthée et al., 2023; Vaulet et al., 2024). These organ-specific cell atlases enable refinement of bulk RNA sequencing analyses. Through *in silico* deconvolution analysis, the composition of intra-graft immune cell types can be quantified and correlated with Banff-defined rejection phenotypes and graft outcomes (Bassaganyas et al., 2024).

## Transcriptomics in pancreas transplantation

Evidence supporting the diagnosis of pancreas graft rejection is mostly derived from histological and immunohistochemistry studies (Drachenberg et al., 2024). Although a pancreas graft biopsy can offer valuable insights into potentially reversible causes of dysfunction, technical difficulties restrict its routine application (Bassaganyas et al., 2024; Aziz et al., 2019; Yoo et al., 2023). Therefore, assessing graft function relies heavily on clinical presentation and biochemical markers in peripheral blood. Published evidence regarding the use of molecular diagnostic techniques in pancreas transplantation remains scarce and primarily focused on the application of dd-cfDNA (Yoo et al., 2023; Ventura-Aguiar et al., 2022; Williams et al., 2022), which, although a promising avenue for detecting established graft injury, has been reviewed elsewhere (Damas et al., 2026).

Pancreas graft tissue molecular analyses rely on techniques and insights derived from studies in other organ transplant populations, mainly kidney and heart. It is expected that some immunological mechanisms involved in pancreas transplant rejection are similar to those in kidney transplant rejection. However, few studies have evaluated pancreas-specific gene expression markers, namely, those associated with either the endocrine or exocrine compartments (Table 1).

**TABLE 1:** Summary of published evidence exploring tissue molecular diagnostics in pancreas transplantation.

Author (Year)	Publication year	Gene analysis technique	Populations (n = size)	Major findings
Luan et al. (2009b)	2009	qRT-PCR with TLDA technology	Different types of acute and chronic rejection (n = 26)	Segregation of samples into two groups associated with clinical outcome; CD20 expression correlates with graft loss
Roufosse et al. (2020)	2020	Nanostring nCounter technology	15 pure ABMR/mixed rejection, 22 TCMR/borderline, 15 no rejection (n = 52)	Identification of a 34-gene set predictive for ABMR
Brown et al. (2025)	2025	Nanostring nCounter technology	Acute TCMR (n = 51)	tCRM scores correlated with increased grade of TCMR and was predictive for treatment resistance

A study published in 2009 advanced the understanding of pancreas allograft rejection through molecular profiling (Luan et al., 2009b). Researchers analyzed gene expression in 26 pancreas transplant biopsies using quantitative real-time polymerase chain reaction (qRT-PCR) using TaqMan Low Density Array (TLDA) technology. The study evaluated the expression of 32 immune-related genes, previously demonstrated in kidney graft rejection, and 10 pancreas-specific genes - concretely six function-related (insulin, amylase, glucagon, somatostatin, cholecystokinin A receptor, and elastase 2A) and four transcription factors involved in regeneration. This molecular analysis revealed two distinct clusters predictive of long-term allograft function. Specifically, the first cluster showed high expression of genes involved in pancreatic function and regeneration, whereas the second cluster was characterized by gene markers associated with acute and chronic rejection. When comparing pancreas graft survival following biopsy as a clinical outcome, researchers found that the second cluster (linked to rejection) was associated with poorer outcomes. Notably, expression of CD20/MS4A1 correlated with graft loss, even in cases where anti-rejection treatment had been administered.

In 2020 Roufosse et al. provided additional insights into the molecular characterization of antibody-mediated rejection (ABMR) in pancreas allografts (Roufosse et al., 2020). They applied a 34-gene signature set using the NanoString™ nCounter (NanoString Technologies), which included endothelial, NK cell, and inflammatory genes derived from genome-wide expression analysis in patients with ABMR in other solid organ transplants (kidney and heart). Furthermore, they suggested that integrating gene expression analysis with donor-specific antibodies (DSA) and histology could improve the prediction of pancreas graft failure. Adding the ABMR 34-gene set score to the other available data at the time of biopsy (DSA and histological features) enhanced the outcome prediction.

Building on these preliminary findings, a 2024 study by Brown et al. aimed to evaluate the applicability of the tissue Common Rejection Module (tCRM) score to identify additional markers of rejection (Brown et al., 2025). This tCRM score, comprising 11 genes, has previously been used as a diagnostic tool to assess the severity of rejection in other solid organ transplant cohorts (Khatri et al., 2013). The study utilized the Nanostring™ Cancer Immune v1.1 oligonucleotide panel on the nCounter platform, enabling multiplex gene expression analysis of both the adaptive and innate immune responses. Using this platform, the researchers analyzed transcripts from 51 pancreas transplant biopsies, comparing gene expression in samples with T cell-mediated rejection (TCMR) to those without histological evidence of rejection. Firstly, they demonstrated the feasibility of using a standardized transcriptomic platform for pancreas graft evaluation, obtaining high-quality RNA from formalin-fixed paraffin-embedded (FFPE) tissue. Secondly, their findings revealed that tCRM scores correlated with increasing rejection severity and were able to distinguish between treatment-resistant and successfully treated cases (Brown et al., 2025).

These studies underscore the value of molecular diagnostic techniques in improving the diagnosis of pancreas graft rejection with greater prognostic accuracy. However, their clinical applicability remains limited due to single-center design, small sample sizes, and the positive selection of biopsy samples. Despite these limitations, the findings reveal unique gene expression patterns distinct from those observed in other transplanted organs, emphasizing the need for further research through larger, multicenter studies to enhance clinical implementation.

## Islet injury in pancreas transplantation

The pathophysiology of diabetes involves a complex interplay of various factors which can induce islet injury, including autoimmunity (as in type 1 diabetes [T1D]), drug interactions, and obesity-associated metabolic stress (Zajec et al., 2022). These insults can lead to inflammation, beta-cell dysfunction or loss, contributing to impaired glucose regulation and diabetes (Cnop et al., 2005). Islet injury and inflammation (insulitis) is seldom observed during acute rejection episodes (Drachenberg et al., 2024). Insulitis can be the hallmark of T1D recurrence after pancreas transplantation, but its incidence is low (Drachenberg et al., 2024). Despite the absence of islet histological abnormalities, there is compelling evidence of the negative impact of acute rejection on graft outcomes (Niederhaus et al., 2013).

The islets of Langerhans are composed of four major cell types: alpha cells (producing glucagon), beta cells (producing insulin), delta cells (producing somatostatin), and gamma cells (also known as PP cells, producing pancreatic polypeptide) (Figure 1). Characterization of islet-specific genes and their functional traits is essential. In 2022 van Gurp et al. published one of the most comprehensive gene sets (n = 260) specific for islet cell types to date (Van Gurp et al., 2022). This standardization addresses the variability introduced by diverse single-cell transcriptomics methods and improves the consistency of results across experimental setups fostering advancements in islet injury.

Most pancreas transplants are performed in patients with T1D, which is a slowly progressive autoimmune disease. During T1D progression, early stages are characterized by innate immune activation, including type I interferon responses and enhanced MHC class I antigen presentation, whereas later stages are marked by T-cell infiltration, upregulation of immune checkpoint molecules such as PDL1 and HLA-E, and the emergence of anti-inflammatory responses (Colli et al., 2020). Building on this understanding, a large-scale single-cell RNA sequencing (scRNA-seq) study analyzing 81,313 transcriptomes from pancreatic islets of 24 organ donors with and without T1D identified IL-32 as a key marker of disease progression, predominantly expressed in activated T cells and natural killer (NK) cells (Fasolino et al., 2022). This study also reported the upregulation of INS, REG1A, and REG3A genes in immune cells from both T1D patients and autoantibody-positive individuals, suggesting early immunological shifts in the pancreatic microenvironment.

In understanding the landscape of diabetes, it is important to recognize the pathways of islet injury that disrupt glucose homeostasis and ultimately lead to insulin insufficiency. These pathways have been studied more extensively in type 2 diabetes (T2D). Although pancreas transplantation is performed predominantly in T1D, selected T2D studies are also relevant because β-cell dysfunction and loss are shared features across diabetes subtypes, and several molecular imaging targets and biomarkers reflect β-cell viability and function independent of disease etiology (Kahn et al., 2021). Unlike the immune-mediated β-cell loss seen in T1D, T2D involves chronic metabolic stress that injures islet cells through non-immune mechanisms. Xin et al. identified distinct subpopulations of beta and alpha cells with differential gene expression patterns among T2D spectrum. Notably, ST8SIA1 and CD9 were identified as markers of specific beta cell populations (Xin et al., 2016). Additional transcriptomic studies, including scRNA sequencing, reveal that T2D β-cells exhibit widespread changes in gene expression, with downregulation of insulin (INS) and exocytosis genes (e.g., STX1A), and upregulation of stress markers such as DDIT3/CHOP and TXNIP (Ngara and Wierup, 2022). Mitochondrial metabolism and protein synthesis pathways are suppressed, while pro-apoptotic and inflammatory pathways are activated. α-cells also show dysfunction, displaying immature, de-differentiated gene profiles. Notably, β-cell loss in T2D is driven more by dedifferentiation and functional impairment than by apoptosis, as many β-cells lose their mature identity marked by the loss of insulin and emergence of progenitor markers like ALDH1A3 without undergoing cell death (Patel and Remedi, 2024).

This refined understanding of both T1D and T2D islet pathology highlights the importance of addressing both immune and metabolic stressors when assessing islet graft injury in pancreas transplantation, particularly donor-related or during acute processes, such as ischemia-reperfusion injury or acute rejection. While immune and metabolic stressors are considered central drivers of islet injury and have driven much of the current molecular diagnostic work, we acknowledge that perioperative islet injury and drug-related toxicity collectively encompassing ischemia reperfusion injury (IRI), machine perfusion-related effects, and immunosuppressive (calcineurin inhibitors, mTOR inhibitors, corticosteroids) associated toxicity could also contribute substantially to early graft dysfunction (Dumbill et al., 2020; Abdelli et al., 2004; Soleimanpour et al., 2010; Barlow et al., 2012; Aylward et al., 2021; Esguerra et al., 2020; Drachenberg et al., 1999).

Pancreas-specific molecular biomarkers may also lead towards the improvement of the diagnosis of pancreas graft injury, as conventional biomarkers are often non-specific. Emerging molecular diagnostic approaches focus on detecting proteins, nucleic acids, or hormones released during islet injury, including glutamate decarboxylase 65 (GAD65), pro-islet amyloid polypeptide (pro-IAPP), the β-cell-selective microRNA miR-375, β-cell free DNA, and the unmethylated/methylated (prepro) insulin promoter ratio (Mirmira et al., 2016). These advancements hold promise for more precise monitoring and targeted interventions.

## Future perspectives for molecular diagnostics in pancreas transplantation

At the most recent Banff meeting in 2022 significant updates were introduced to the classification, specifically the guidelines for TCMR, ABMR, and islet pathology in pancreas transplantation. The meeting report also highlighted the potential of non-invasive diagnostic methods, reflecting the increasing focus on molecular approaches to improve the diagnosis of pancreatic transplant rejection (Drachenberg et al., 2024). More broadly, across solid-organ transplantation, molecular diagnostics are increasingly explored to complement conventional histology and provide mechanistic insight into graft injury and rejection processes (Loupy et al., 2017; Naesens and Sarwal, 2010; Lazarou et al., 2025). Although their implementation in pancreas transplantation remains limited, these approaches hold considerable promise for improving diagnostic precision.

The study by Brown et al. demonstrated that molecular studies can be effectively performed on pancreas graft FFPE samples using the NanoString™ platform (Brown et al., 2025). This paves the way for the B-HOT gene panel’s application in pancreas graft biopsies. The Nanostring™ nCounter platform has potential for on-site clinical applicability, whereas maintaining the possibility for centralized multicenter data analysis and the development of predictive algorithms (Zielinski et al., 2025). Using machine learning, these algorithms could further enhance the molecular classification of acute rejection and potentially refine the current Banff classification (Zielinski et al., 2025). However, the diagnostic performance of transcriptomic classifiers depends on the selection of gene panels and analytical approaches, although the development of the B-HOT panel was a major step in the right direction however, consensus criteria for pancreas-specific implementation are still evolving. Continued multicenter validation and harmonization of molecular platforms will therefore be important for broader clinical implementation (Loupy et al., 2017; Adam and Mengel, 2015).

While there is enthusiasm about its potential clinical application, it is crucial to acknowledge the unique characteristics of pancreas graft tissue. Notably, the B-HOT panel was designed without incorporating pancreas graft-specific genes. The pancreas consists of two distinct compartments, exocrine and endocrine, each of which may respond differently to injury, impacting graft outcomes. Although several molecular signatures of rejection have been identified in other transplanted organs, organ-specific validation remains essential, as differences in tissue architecture and cellular composition may influence injury responses in the pancreas (Loupy et al., 2017; Halloran et al., 2023). To better understand these effects, studies utilizing unsupervised gene analysis, such as bulk RNA sequencing (RNA-seq) or single-cell RNA sequencing (scRNA-seq), are needed to identify the molecular patterns of pancreas graft rejection. The impact of identified pancreas-specific genes could be further corroborated in a clinically suitable platform, such as the NanoString™ nCounter, which allows the addition of custom genes to the B-HOT panel.

The next step is to map the spatial distribution of key cells involved in graft outcomes. As previously demonstrated for kidney grafts (Lamarthée et al., 2023), scRNA-seq could facilitate the development of a comprehensive pancreas graft cell atlas. Spatial transcriptomics, which identifies cell location within the graft tissue, may further improve our understanding of cell–cell interactions and molecular pathways involved in graft injury (Li et al., 2023; Martin-Martin et al., 2024). Although these high-throughput technologies present exciting opportunities, practical considerations remain. Transcriptomic analyses require sufficient tissue and high-quality nucleic acids, which can be challenging when working with small pancreas biopsy samples. Nevertheless, ongoing advances in targeted multiplex platforms and protocols optimized for FFPE tissue are increasingly enabling the application of molecular diagnostics in routine clinical specimens (Adam and Mengel, 2015; Fang et al., 2020).

It is also important to recognize that transcriptional activity does not necessarily equate to functional protein expression. Despite the abundance of data generated through transcriptomic analyses, molecular findings should ideally be corroborated by complementary techniques such as immunofluorescence, immunohistochemistry, or proteomic approaches. Emerging proteomic and metabolomic strategies may further enhance the characterization of graft injury by identifying circulating or tissue biomarkers associated with immune activation. Although still largely investigational in pancreas transplantation, studies in other solid-organ transplants highlight their potential for improving non-invasive monitoring and mechanistic understanding of rejection processes (Christians et al., 2016).

In conclusion, molecular studies in pancreas transplantation remain scarce but are essential for refining the diagnosis of graft-specific acute rejection. Furthermore, in an organ in which graft biopsies are infrequently performed, a deeper understanding of tissue injury at the molecular level may facilitate the development of pancreas-specific non-invasive biomarkers and improve diagnostic accuracy. Pancreas graft biopsy itself presents several challenges, including procedural risk, limited tissue yield, and variability in interpretation between centers (Singh et al., 2024; Par et al., 2017). These limitations highlight the potential value of integrating histologic assessment with molecular profiling and emerging circulating biomarkers to develop a more comprehensive and precise diagnostic framework for pancreas graft rejection.
