# Supplementary material for: Molecular diagnostics in pancreas transplantation: past, present, and future
Source: Front Mol Biosci. 2026 Jun 22;13:1825364. doi: 10.3389/fmolb.2026.1825364 (PMC13333494; doi:10.3389/fmolb.2026.1825364)
Supplement: Supplementary file 1 [file Table1.docx]

| **Author**  **[Ref]** | | **Publication Year** | | **Gene analysis technique** | **Populations (n=size)** | **Major findings** |
| --- | --- | --- | --- | --- | --- | --- |
| Luan et al.  [38] | | 2009 | | qRT-PCR with TLDA technology | Different types of acute & chronic rejection (n=26) | Segregation of samples into two groups associated with clinical outcome; CD20 expression correlates with graft loss |
| Roufosse et al.  [39] | | 2020 | Nanostring nCounter technology | 15 pure ABMR/mixed rejection, 22 TCMR /borderline, 15 no rejection (n=52) | Identification of a 34-gene set predictive for ABMR |  |
| Brown et al.  [40] | | 2025 | | Nanostring nCounter technology | Acute TCMR (n=51) | tCRM scores correlated with increased grade of TCMR and was predictive for treatment resistance |
